# Effectiveness and safety of acupuncture for Parkinson’s disease anxiety: a systematic review and meta-analysis

**DOI:** 10.3389/fnagi.2025.1663059

**Published:** 2025-10-15

**Authors:** Lu Chen, Hong-xiao Xu, Zhao-qin Wang, Guo-na Li, Lu-yi Wu, Yan Huang, Huan-gan Wu, Jian-hua Zhou

**Affiliations:** ^1^Shanghai Eighth People's Hospital, Shanghai, China; ^2^Shanghai University of Traditional Chinese Medicine, Shanghai, China; ^3^Yueyang Hospital of Integrative Medicine, Shanghai University of Traditional Chinese Medicine, Shanghai, China; ^4^Shanghai Institute of Acupuncture and Meridian Research, Shanghai, China

**Keywords:** acupuncture, Parkinson’s disease, anxiety, meta-analysis, systematic review

## Abstract

**Background:**

Individuals with Parkinson’s disease (PD) commonly experience anxiety, with a prevalence of 31%. This study systematically evaluates the efficacy and safety of acupuncture for anxiety related to PD.

**Method:**

Nine databases were searched for randomized controlled trials (RCTs) published from inception to August 24, 2025. RCTs comparing acupuncture and moxibustion treatments (with or without other therapies, e.g., western medicine, routine care, sham acupuncture) to other therapies alone for managing PD anxiety were included. Data were analyzed using the R software (version 4.5.1). In accordance with PRISMA-2020 guidelines, two reviewers independently extracted data and assessed the risk of bias using the Cochrane risk of bias tool (ROB 2.0). The certainty of the evidence was graded using the GRADE (Grading of Recommendations Assessment, Development, and Evaluation) according to GRADE handbook.

**Results:**

A total of 10 studies were included, comprising 1,000 patients with anxiety after PD. The meta-analysis indicated that, compared to the control group, the acupuncture group showed significant improvements in HAMA and SAS scores (SMD = −3.64, 95% CI [−5.06 to −2.23]; SMD = −7.76, 95% CI [−10.10 to −5.41]), as well as significant improvements in HAMD and SDS scores (SMD = −2.93, 95% CI [−4.25 to −1.60]; SMD = −8.35, 95% CI [−8.88 to −7.82]). The reported adverse events related to acupuncture were minimal and less severe.

**Conclusion:**

Acupuncture can successfully reduce anxiety symptoms in PD patients. Additional higher quality randomized controlled trials are required to ascertain the safety and effectiveness of acupuncture as a therapy for anxiety in PD patients.

**Systematic review registration:**

https://www.crd.york.ac.uk/PROSPERO/, Identifier CRD42024601125.

## 1 Introduction

Among neurodegenerative (GBD 2016 Parkinson's Disease Collaborators, 2018) diseases, Parkinson’s disease (PD) is the second most prevalent, and forecasts indicate that its incidence will likely treble within the next three decades (Tansey et al., 2022; Tolosa et al., 2021). Neuropsychiatric manifestations are commonly observed in individuals with PD throughout the course of the disease (Weintraub et al., 2022). It is common for non-motor signs of PD, such as hyposmia, sleep problems (Gros and Videnovic, 2020; Iranzo et al., 2024), depression, and constipation (Schapira et al., 2017), to appear by many years before the motor symptoms of the disease. Among these non-motor symptoms (Chaudhuri et al., 2006), anxiety is particularly prominent and typically lacks effective treatment options. Anxiety in PD not only exacerbates the burden on caregivers but also worsens motor symptoms, increases the fear of falling, and may contribute to cognitive deterioration (Aarsland et al., 2021; Jellinger, 2022) decline (Dissanayaka et al., 2010). For those affected by PD, anxiety has been shown to have a more profound negative impact on quality of life compared to depression. Individuals with PD commonly experience anxiety, with a prevalence of 31% (Ascherio and Schwarzschild, 2016; Ben-Shlomo et al., 2024; Carey et al., 2021; Tysnes and Storstein, 2017), approximately twice that of the general population (15%) (Broen et al., 2016). Moreover, gait disturbances and freezing of gait have been associated with anxiety symptoms. Consequently, anxiety should be recognized as a significant symptom in PD, particularly in the context of dyskinesia (Mirelman et al., 2019).

The prevalence of anxiety in patients with Parkinson’s disease (20–46%) is higher than that of depression (17% for major depression, 22% for mild depression), and anxiety exerts a more pronounced negative impact on quality of life by directly impairing motivation, treatment adherence, and cognitive function (Reijnders et al., 2008; Opara et al., 2012; Ehgoetz Martens et al., 2016). However, there are only a few treatment modalities for anxiety in PD (Martens et al., 2016; Tolosa et al., 2006). Cognitive behavioral therapy (CBT) has been identified as an effective nonpharmacological intervention for depression and anxiety in PD (Cook et al., 2017; Kwok et al., 2019; Mak et al., 2017; Vijiaratnam et al., 2021). Despite its efficacy, the high cost of CBT has led to reduced patient adherence (Pachana et al., 2013). Emerging evidence suggests that acupuncture may be comparable in efficacy to CBT (Chae et al., 2008). Considering acupuncture’s treatment duration, time commitment, and cost, it may be more widely accepted for PD’s treatment in China. Given the situation of current treatments for anxiety in PD, there is an increasing demand in Western societies to investigate effective alternative therapies that offer high feasibility and fewer adverse effects. According to clinical guidelines, acupuncture and moxibustion are recommended as a Grade B treatment intervention for addressing the mental symptoms of PD, potentially supplementing or replacing existing therapeutic approaches (Cho et al., 2018; Noh et al., 2017; Weintraub et al., 2022). Randomized controlled trials (RCTs) of acupuncture in PD have been published successively (Li et al., 2023). Acupuncture is believed to ameliorate motor symptoms and associated anxiety by stimulating specific acupuncture points, which in turn may modulate the balance of neurotransmitters within the central nervous system, such as by increasing dopamine release. Additionally, acupuncture may facilitate the release of inhibitory neurotransmitters like gamma-aminobutyric acid (GABA), potentially reducing anxiety levels (Chen et al., 2020). However, the quality of these studies varies, which can be a disadvantage for clinicians seeking high-quality evidence and potential treatment strategies.

This article intends to establish a framework for clinical decision-making and compile pertinent randomized controlled studies to assess the feasibility and safety of acupuncture as a treatment for anxiety in PD. The assessment is guided by the principles of evidence-based medicine (Heneghan et al., 2017).

## 2 Methods

### 2.1 Literature search strategy

The Preferred Reporting Items for Systematic Reviews and Meta-Analyses (PRISMA) guidelines (Page et al., 2021) were followed during the whole process of this systematic review and meta-analysis. This study protocol has been assigned the registration number CRD42024601125 by the PROSPERO system.

Nine databases were searched electronically: Web of Science, Scopus, China National Knowledge Infrastructure (CNKI), Chinese Biomedical Database, VIP Journal Integration Platform, PubMed, Embase, Cochrane Library, and Wanfang Database. Between the creation of each database and August 24, 2025, studies were found and vetted. EndNote (version 20) was utilized to import the references, facilitating the exclusion of duplicate publications. Two experienced researchers (CL and XHX) independently performed the literature screening based on the established inclusion and exclusion criteria. The researchers first examined the titles and abstracts of the literature, omitting those that did not meet the inclusion criteria. Subsequently, full-text articles were retrieved and examined to verify that their content satisfied the inclusion criteria. The researchers then cross-validated the materials they had individually included and excluded. In cases of disagreement, a third-party researcher, HY, was consulted to make the final determination. The Supplementary Table S1 provides detailed information on the search strategies employed.

### 2.2 Inclusion and exclusion criteria

The full texts of the selected articles were downloaded and meticulously reviewed by the research team to ascertain their eligibility for inclusion. For the purpose of this analysis, we focused on clinical studies, which could be either prospective. The eligibility criteria were established based on the PICOS framework which stands for population, intervention, comparison, outcome, and study type. This includes the following: (1) Patients were given a conclusive diagnosis of anxiety related to PD in line with the MDS Clinical Diagnostic Criteria for PD, 2015 edition. No restrictions were placed on age, gender, race, nationality, or the duration of illness. (2) The experimental group was treated with acupuncture or moxibustion, and they had the option of receiving additional treatments (such as western medicine, routine care). (3) The control group was treated with alternative therapies, excluding acupuncture and moxibustion (such as western medicine, routine care, or sham acupuncture). (4) The outcomes assessed included the Hamilton Depression Rating Scale (HAMD), Hamilton Anxiety Scale (HAMA), Self-Rating Anxiety Scale (SAS), and Self-Rating Depression Scale (SDS). (5) The investigations were RCTs.

The following were the conditions for an exclusion: (1) studies were not RCTs; (2) studies with populations that were not clearly identified as PD anxiety patients or had unclear diagnoses; (3) studies where the control group received acupuncture treatments; (4) duplicate publications; and (5) studies that failed to report the Anxiety Scale or presented incomplete data.

### 2.3 Data extraction

The two researchers read the literatures and extract the following information based on the predetermined criteria: (1) the author and the year was published, (2) number of samples, (3) average age, (4) treatment intervention measures (acupoints), (5) control group, (6) duration (treatment course, disease course), (7) main outcomes, and (8) negative outcomes. The Grading of Recommendations Assessment, Development, and Evaluation (GRADE) methodology were used to assess the quality of the evidence (Guyatt et al., 2008), which is a systematic approach for evaluating the quality of evidence. Two researchers (CL and XHX) independently assessed the quality of the included studies. Disagreements in the evaluation were resolved through discussion, or, if consensus was unattainable, by involving a third assessor (HY).

### 2.4 Quality assessment

Two reviewers used the Cochrane ROB2 tool to independently assess the risk of bias. The Cochrane Risk of Bias tool (Sterne et al., 2019) was utilized in order to evaluate the potential for bias in the studies that were included in the review. The reviewers. LGN and WLY conducted an independent analysis of the ROB2 in the studies that were included. Their analysis focused on six key areas: (1) the randomization process; (2) variations from the planned interventions; (3) missing outcome data; (4) outcome assessment; (5) selection of reported outcomes; and (6) overall bias. It was decided to seek the advice of HY, a third reviewer, in order to address any inconsistencies that surfaced throughout the inspection.

### 2.5 Strategy for data synthesis

Meta-analyses were performed when the studies exhibited comparability and employed uniform outcome measures. Data analysis employed random-effects models, each with a 95% confidence interval (CI). The R software (version 4.5.1)[Meta] package were utilized for the meta-analysis of the data as necessary. The I^2^ statistic was used to determine the degree of heterogeneity among the studies. Publication bias was evaluated through a funnel plot (Salanti et al., 2011), and Egger’s test was utilized when ten or more studies were present. The assessment of statistical heterogeneity among the studies utilized the I^2^statistic, categorized into three levels: low heterogeneity (I^2^ < 50%), moderate heterogeneity (I^2^ = 50–74%), and high heterogeneity (I^2^ ≥ 75%) (Higgins et al., 2003). In cases of significant heterogeneity, a qualitative synthesis of the data will be conducted to investigate potential sources of variability and to offer a narrative interpretation of the results.

## 3 Results

### 3.1 Description of included trials

Through our thorough search technique, we were able to identify a total of 480 articles. The dataset was reduced to 193 articles after duplicates were removed. Upon screening the titles and abstracts, 147 articles were excluded for various reasons: 38 were reviews, 26 were case reports, 18 were duplicates, 33 involved inappropriate interventions, and 32 were unrelated to the disease in question.

The complete texts of the remaining articles were subsequently downloaded and thoroughly evaluated by two researchers (CL and XHX). During this process, 16 articles were excluded as they did not adhere to the RCT design. An additional 16 articles were excluded due to non-compliance with the disease criteria. Furthermore, 14 articles were excluded because their control groups included acupuncture treatment.

**Figure 1:**
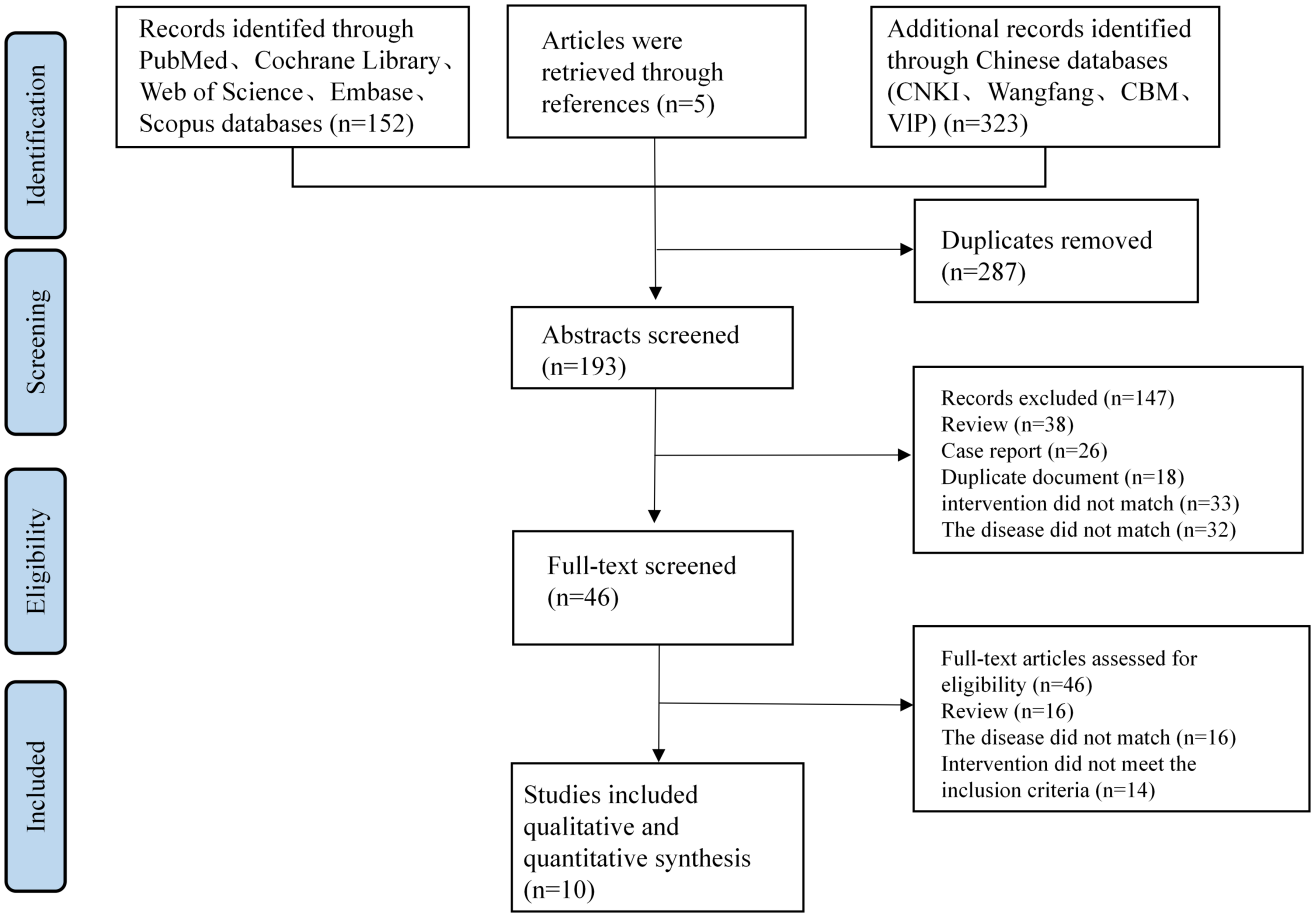
PD anxiety’s search and filtering flowchart.

Following the application of our inclusion and exclusion criteria, a total of 10 publications (Fan et al., 2022; Lu, 2022; Liu, 2020; Ma et al., 2024; Xu and Xia, 2017; Zhu et al., 2025; Li and Shi, 2025; Bai and Wang, 2021; Li et al., 2021; Song, 2021) were selected for inclusion (see Table 1). These publications reported data from 1,000 patients aged 46 to 89 years with PD anxiety (see Figure 1). All included studies were prospective studies.

**Table 1:** Baseline characteristics of included reviews.

Researcher (year)	Sample size (T/C, n)	Mean age ± SD (T/C)	Intervention (treatment/control)	Duration	Outcome measures
Xu and Xia (2017)	70 (35/35)	71.8 ± 5.3/73.2 ± 4.9	Routine care + Auricular acupressure/Routine care	9 days	SAS, SDS
Liu (2020)	60 (30/30)	54.55 ± 1.21/59.31 ± 13	Medication + Electroacupuncture/Medication alone	8 weeks	HAMD, HAMA
Lu (2022)	64 (32/32)	61.37 ± 1.61/63.78 ± 1.13	Body acupuncture/Sham acupuncture	4 weeks	HAMA
Fan et al. (2022)	64 (32/32)	61.03 ± 9.80/62.66 ± 6.94	Body acupuncture/Sham acupuncture	8 weeks	HAMA
Ma et al. (2024)	300 (150/150)	72.31 ± 6.52/71.29 ± 6.81	Empathic care + Acupoint application/Routine care	15 days	HAMD, HAMA
Li et al. (2021)	100 (50/50)	61.56 ± 7.51/62.49 ± 7.53	Medication + Electroacupuncture/Medication alone	8 weeks	SAS, SDS
Song (2021)	124 (62/62)	72.64 ± 2.50/72.53 ± 2.56	Rehabilitation exercise+ Body acupuncture / rehabilitation exercise	1 month	HAMD, HAMA
Bai and Wang (2021)	58 (29/29)		Medication + Body acupuncture/Medication alone	2 weeks	HAMA
Li and Shi (2025)	100 (50/50)	58.49 ± 2.68/58.23 ± 3.25	Medication + Body acupuncture/Medication alone	2 months	SAS, SDS
Zhu et al. (2025)	60 (30/30)	65.2 ± 8.3/64.8 ± 7.9	Medication + Body acupuncture/Medication alone	12 weeks	HAMD, HAMA

### 3.2 Risk of bias

**Figure 2:**
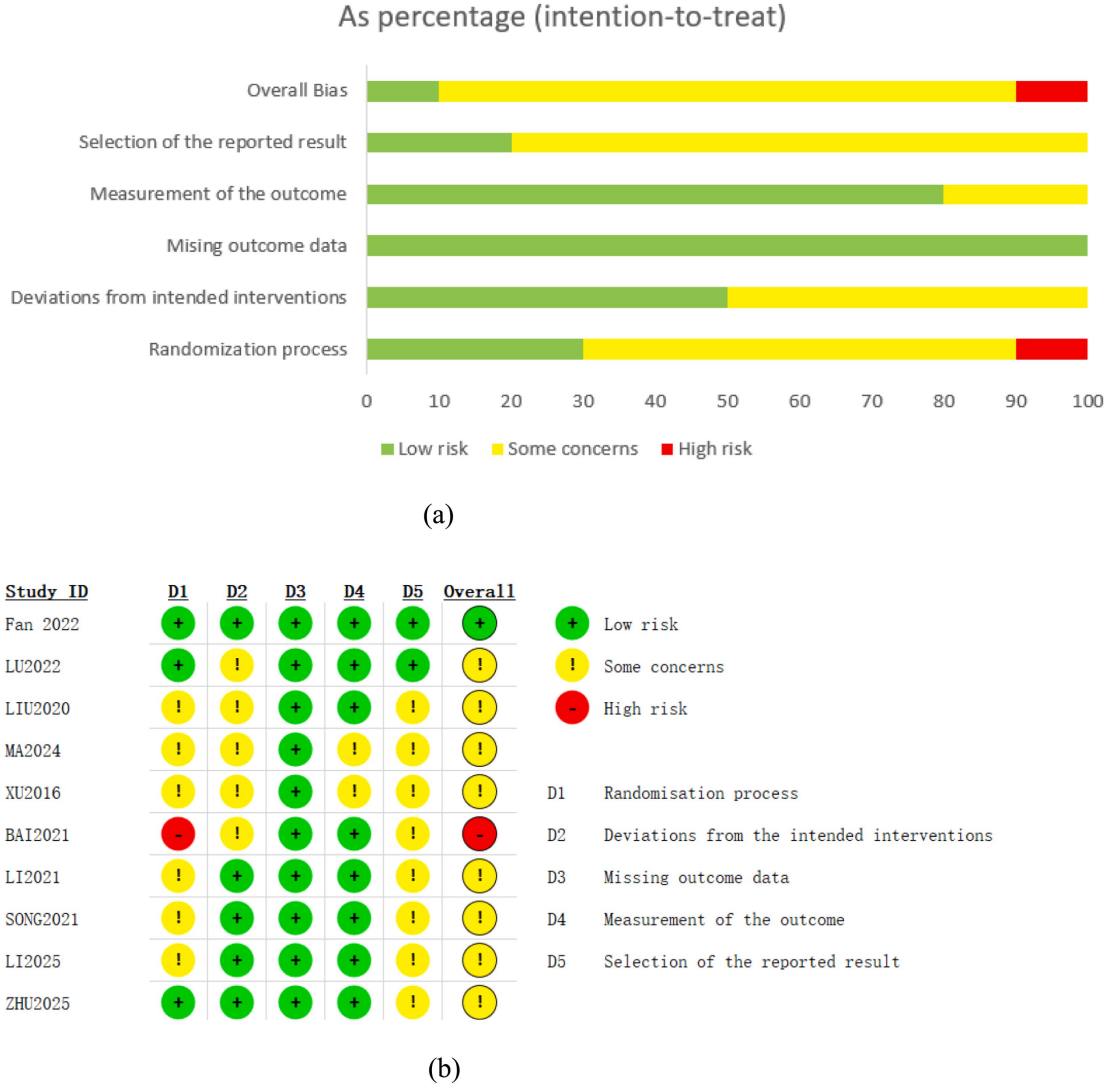
Assessment of risk of bias. **(a)** Risk of bias graph; **(b)** Risk of summary.

According to the ROB 2.0 assessment, eight (Lu, 2022; Liu, 2020; Ma et al., 2024; Xu and Xia, 2017; Zhu et al., 2025; Li and Shi, 2025; Li et al., 2021; Song, 2021) out of the 10 included studies were rated as having some concerns, one study (Fan et al., 2022) was judged to be at low risk of bias, and one (Bai and Wang, 2021) was classified as being at high risk of bias. Three papers (Fan et al., 2022; Lu, 2022; Zhu et al., 2025) provided comprehensive explanations of the randomization procedure and technique. In the randomization process, one study (Bai and Wang, 2021) was rated as high risk because it used an odd-even allocation method based on the order of presentation. Among the 10 studies, three (Fan et al., 2022; Lu, 2022; Zhu et al., 2025) described allocation concealment using sealed opaque envelopes that gave a thorough explanation of the measures employed for blinding. In terms of participant and personnel blinding, two studies (Lu, 2022; Fan et al., 2022) employed a sham-needle double-blind design, while two studies (Zhu et al., 2025; Fan et al., 2022) utilized a third-party assessment blinding method. The other seven studies did not specifically discuss blinding, which raised some worries about the possibility of bias. The 10 studies showed that there was a very minimal risk of bias due to inadequate outcome data. This was due to the fact that either the data were full or any missing data were not sufficient to materially change the effect estimate. Two trials (Fan et al., 2022; Lu, 2022) were identified that offered pre-registration alternatives and were assessed to have a low risk of bias. Failure to pre-register may lead to reporting bias, be rated as having some concerns of bias (see Figure 2).

### 3.3 The forest of outcome PD anxiety

**Figure 3:**
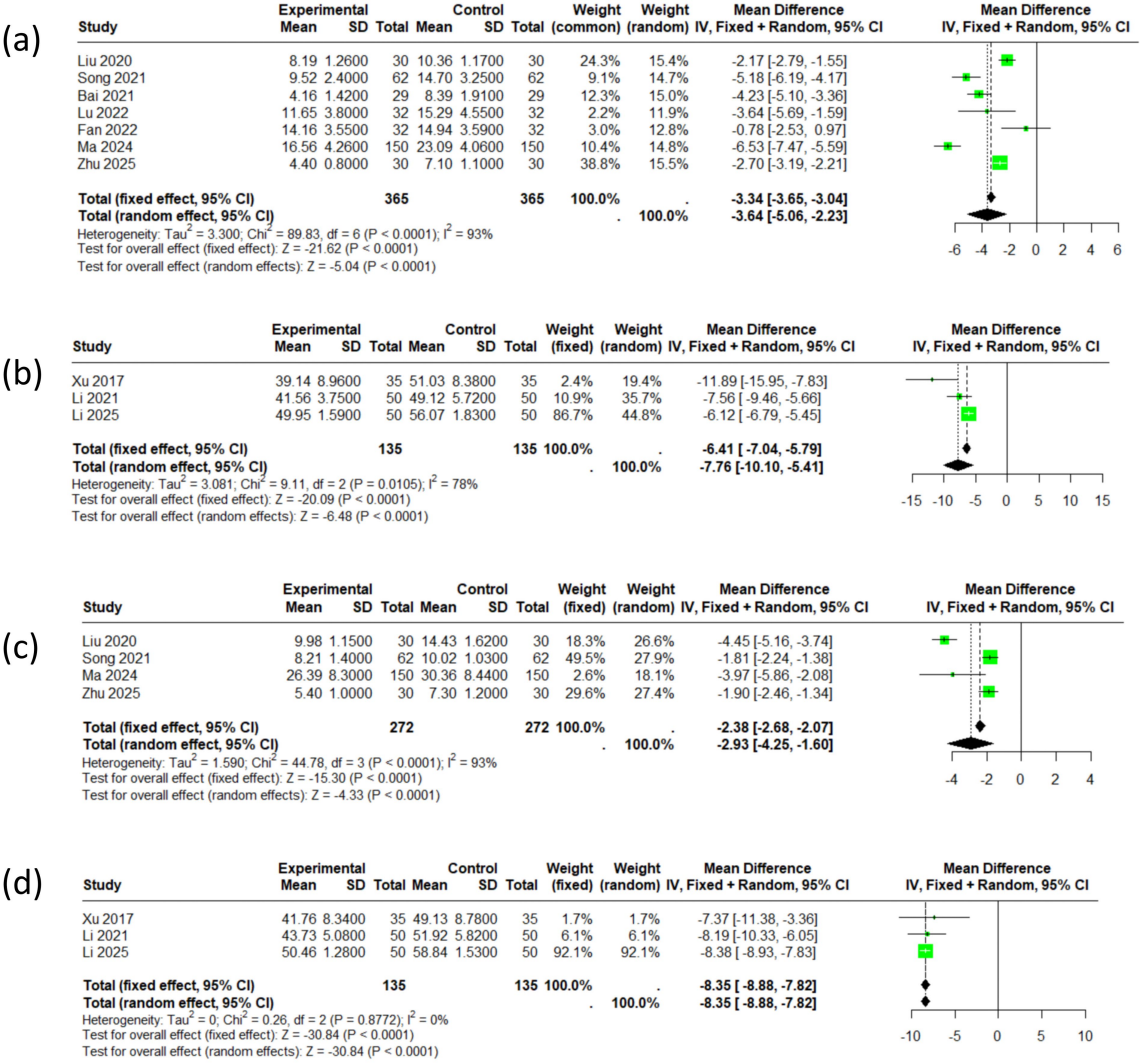
The forest of the outcomes (**a**: HAMA, **b**: SAS, **c**: HAMD, **d**: SDS).

Seven articles reported the total HAMA scores, encompassing a cohort of 730 patients. The selected effect size metric is the standardized mean difference (SMD). I^2^ is 93%, indicating significant heterogeneity among the studies. The meta-analysis revealed an SMD of −3.64 (95% CI: −5.06 to −2.23), and the corresponding forest plot diamond was positioned to the left of the null hypothesis line. Three articles reported the total SAS scores, encompassing a cohort of 270 patients. The meta-analysis revealed an SMD of −7.76 (95% CI: −10.10 to −5.41). Comprehensive analysis indicates that acupuncture therapy has statistically significant positive effects on anxiety symptoms (see Figures 3a,b).

Four articles reported on the HAMD and included a total of 544 patients. The impact size metric that was chosen is the standardized mean difference (SMD). I^2^ is 93%, indicating significant heterogeneity among the studies. The meta-analysis indicated an SMD of −2.93 (95% CI: −4.25 to −1.60), and the forest plot diamond was positioned to the left of the null hypothesis line, suggesting a favorable effect of acupuncture. Three articles reported the total SDS scores, encompassing a cohort of 270 patients. The meta-analysis revealed an SMD of −8.35 (95% CI: −8.88 to −7.82). In conclusion, the intervention group exhibited significantly improved efficacy in treating depressive symptoms in individuals with PD (see Figures 3c,d).

### 3.4 The subgroup analysis of Parkinson’s disease anxiety

#### 3.4.1 HAMA score

**Figure 4:**
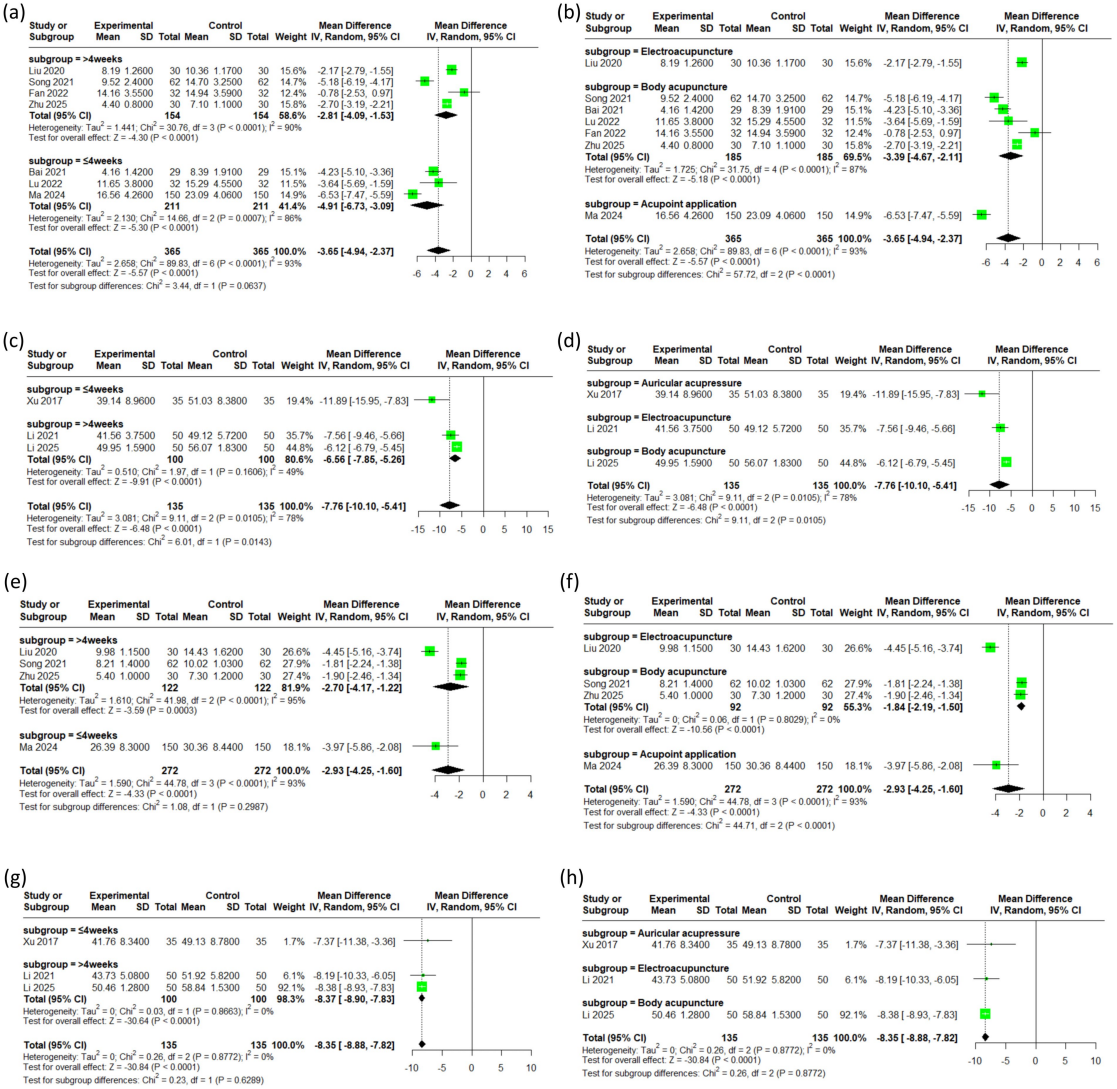
The subgroup analysis of the outcomes (**a,b**: HAMA, **c,d**: SAS, **e,f**: HAMD, **g,h**: SDS).

For the HAMA outcomes, the I^2^ value was 86 within the first 4 weeks of treatment and 90 beyond 4 weeks, compared to an overall I^2^ of 93 before subgrouping, indicating a modest reduction in heterogeneity. For different acupuncture modalities, the I^2^ value decreased from 93 to 87, indicating a reduction in heterogeneity (see Figures 4a,b).

#### 3.4.2 SAS score

Compared to the pre-subgrouping overall I^2^ of 78, the I^2^ for SAS outcomes beyond 4 weeks was lower at 49, indicating some reduction in heterogeneity (see Figures 4c,d).

#### 3.4.3 HAMD score

For the HAMD outcomes, the I^2^ value plummeted from 93 to 0 after subgroup analysis by acupuncture type, confirming that the high heterogeneity was primarily attributable to the specific acupuncture intervention (Figures 4g,h). In contrast, the I^2^ value remained largely unchanged when analyzed by treatment duration (see Figures 4e,f).

### 3.5 The sensitivity analysis of Parkinson’s disease anxiety

**Figure 5:**
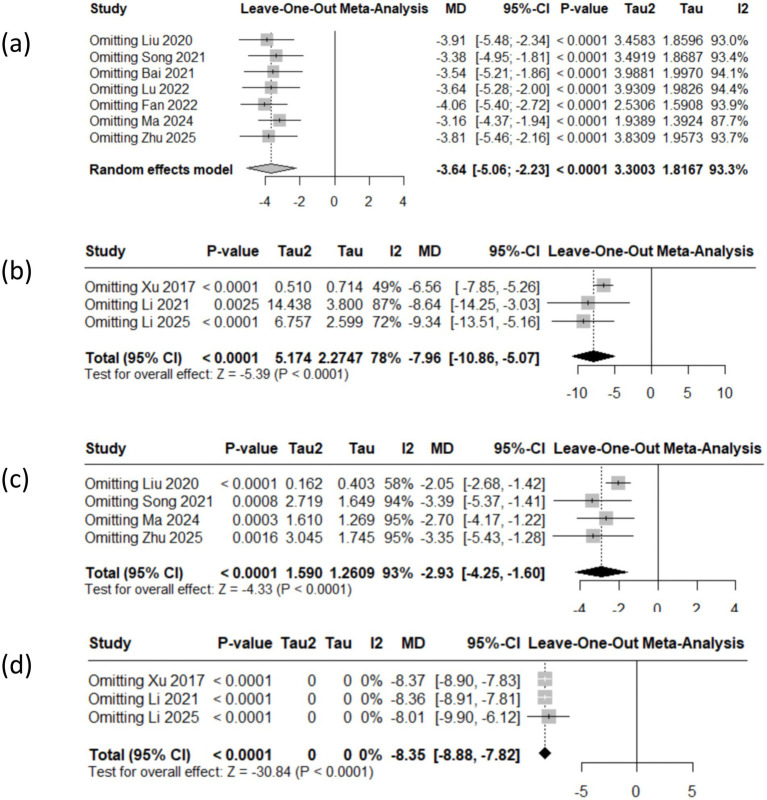
The sensitivity analysis of the outcomes (**a**: HAMA, **b**: SAS, **c**: HAMD, **d**: SDS).

The reliability of the results in this study was validated through sensitivity analysis, which entailed the sequential exclusion of each study to evaluate the stability of the effects. The aggregated findings for the HAMA, SAS, HAMD and SDS scores exhibited consistent effects, the point estimate falls within the confidence interval, outcomes exhibit robustness (see Figure 5).

### 3.6 Publication bias

Formal testing for publication bias was not performed owing to the limited quantity of studies available per measure.

### 3.7 GRADE evidence profile for the studies in the meta-analysis

According to the GRADE approach, the quality of evidence was rated as high for HAMA, moderate for SAS and HAMD, and low for SDS (see Table 2).

**Table 2:** GRADE evidence profile for the studies in the meta-analysis.

Outcome	No. study	No. patients	Certainty assessment					Summary of findings	
			Risk of bias	Inconsistency	Indirectness	Imprecision	Publication bias	Effect size	Certainty
								Pooled MD (95% CI)	
HAMA	7	730	Serious^a^	Serious^b^	NS	NS	NA	−3.64 [−5.06, −2.23]	Low
SAS	3	270	NS	Serious^b^	NS	NS	NA	−7.76 [−10.10, −5.41]	Moderate
HAMD	4	544	NS	Serious^b^	NS	NS	NA	−2.93 [−4.25, −1.60]	Moderate
SDS	3	270	NS	NS	NS	NS	NA	−8.35 [−8.88, −7.82]	High

a All included studies were assessed using the Cochrane Risk of Bias tool (version 2.0), and some were judged to be at high risk of bias.

b The important heterogeneity was found.

No., number; CI, confidence interval; NS, not serious; NA, not available; MD, mean difference.

### 3.8 Adverse effects

We extracted all safety events from the RCTs. A total of five documents mentioned adverse reactions after treatment. Among them, mild adverse reactions related to acupuncture included redness and swelling after needle insertion, hematoma, pain, needle retention, and fear of needles (Resting and drinking warm water can help alleviate the symptoms). Other adverse reactions included nausea and vomiting, hypotension, hallucinations, movement disorders, constipation, dizziness, headache, and abnormal liver function. However, due to incomplete reporting in the original documents, the causal relationship could not be fully determined (see Table 3).

**Table 3:** Adverse reactions included in the literature.

Safety data	Experimental group	Control group
Fan et al. (2022)	Hematoma (2/32); Needle retention (2/32)	/
Zhu et al. (2025)	Pain at the acupuncture site (3/30), local redness and swelling after acupuncture (3/30), fear of needles (3/30); Nausea (4/30), vomiting (3/30), hypotension (2/30), hallucinations (1/30), dyskinesia (2/30)	Nausea (5/30), vomiting (2/30), hypotension (1/30), hallucinations (2/30), dyskinesia (3/30), abnormal liver function (1/30)
Ma et al. (2024)	Nausea (3/150), constipation (3/150), dizziness (5/150)	Nausea (12/150), constipation (8/150), dizziness (7/150)
Lu (2022)	Hematoma (2/32); Needle retention (2/32)	/
Liu (2020)	Stomach discomfort (0/30); Nausea and vomiting (1/30); Headache (1/30)	Stomach discomfort (2/30); Nausea and vomiting (2/30); Headache (2/30)

## 4 Discussion

This comprehensive review and meta-analysis of randomized controlled trials (RCTs) demonstrates that acupuncture is both safe and effective for treating anxiety in people who have PD. This study assesses the effectiveness and safety of acupuncture and moxibustion in reducing anxiety symptoms related to PD. The evaluation is based on 10 RCTs. According to the data, acupuncture is a good way to manage anxiety in this demographic, and there have not been any serious adverse effects reported.

Our meta-analysis indicates that multiple acupuncture interventions, such as acupuncture alone, the combination of western medicine and acupuncture, empathic care with acupressure, and auricular pressure point stimulation, administered over durations of 9 to 84 days, resulted in significant improvements in HAMA/SAS scores among patients experiencing anxiety related to PD, relative to control groups. Despite these findings, the evidence regarding the effectiveness of acupuncture in alleviating specific anxiety symptoms, including excessive worry, fear, and fatigue, is inadequate. The significant heterogeneity observed across the studies in this review underscores the necessity for additional research employing more rigorous methodologies to confirm these preliminary findings.

This substantial heterogeneity might stem from the variability in acupuncture techniques across studies, including variances in treatment dose and frequency, acupoint selection, the skill level of session length. Additionally, it could be attributed to the wide age range and diverse levels of anxiety among participants. Previous meta-analyses have frequently highlighted the pervasive heterogeneity in acupuncture treatments. Even people with the same ailment may have various homeostatic imbalances, according to traditional Chinese medicine’s theoretical framework. This has turned into one of the most challenging issues in the systematic review and distribution of acupuncture research (White et al., 2008). It is worth noting that standardization of acupuncture practices is still a topic of debate.

The subgroup analyses in this study revealed several interesting findings. Treatment duration and acupuncture type may be key factors influencing therapeutic efficacy; however, these results should be interpreted with caution. First, subgroup analyses are observational in nature and may be affected by confounding factors. As all included patients were elderly, they may have had more comorbidities, and the observed differences may not be entirely attributable to treatment duration or specific acupuncture techniques. Second, the limited number of studies in certain subgroups may have resulted in insufficient statistical power, increasing the risk of false-positive or false-negative outcomes. Therefore, these subgroup findings should be regarded as exploratory and hypothesis-generating rather than conclusive.

The Cochrane Risk of Bias Assessment Tool (Higgins et al., 2011) notes that failure to implement blinding of investigators/participants may lead to an overestimation of effect sizes (on average approximately 15–20%). The unique nature of acupuncture procedures (e.g., the sensation of deqi) makes complete blinding difficult to achieve, but the absence of assessor blinding amplifies measurement bias in subjective scales (e.g., HAMD/SDS). Only two of the 10 studies (Fan et al., 2022; Zhu et al., 2025) were pre-registered in the China Clinical Trial Registry, while the remaining studies carry a risk of protocol deviation, which undoubtedly increases the risk of selective reporting (e.g., hiding negative results or adjusting the analysis protocol).

This study’s GRADE evidence quality assessment demonstrated considerable variation in the strength of evidence among the different outcome measures. The HAMA was rated as high quality, providing strong confidence that the estimated effect of acupuncture on reducing anxiety is reliable. In contrast, both the SAS and HAMD were graded as moderate, indicating that while the current results are likely valid, further studies could still impact these conclusions. Heterogeneity in these outcomes may stem from differences in study design or intervention details. Some studies used routine care as the control group (non-active control), which may have overestimated the efficacy of acupuncture. It is recommended to emphasize preliminary evidence rather than definitive conclusions. The SDS was assessed as low quality, implying limited confidence in the effect estimates, likely due to risk of bias, imprecision, or inconsistency among studies. Thus, findings related to SDS should be considered exploratory and require validation through more rigorous trials.

Acupuncture can reduce the activation of the cortex and limbic system (Lin et al., 2023; Xu et al., 2023) in PD patients, areas associated with emotion regulation and anxiety. By decreasing the hyperactivation of these regions, acupuncture may alleviate anxiety symptoms (Deuel and Seeberger, 2020). Acupuncture has the potential to reduce anxiety symptoms through its effects on the hypothalamic–pituitary–adrenal (HPA) axis (Zheng et al., 2024), leading to decreased secretion of stress hormones like cortisol. Acupuncture may reduce anxiety symptoms by modulating serotonin (5-HT) and norepinephrine (NE) levels (Lu et al., 2022), as these neurotransmitters are essential for mood regulation (Höglinger et al., 2024; Tan et al., 2020).

The limitations of this review primarily stem from the inherent shortcomings of the included studies. As the meta-analysis was based on a limited number of studies, the generalizability of the conclusions is constrained, statistical power is reduced, and the strength of evidence (affecting the GRADE assessment) is diminished. The use of subjective scales as outcome measures may introduce reporting biases due to their susceptibility to subjective interpretation. Furthermore, individual researchers may have brought their own perspectives when interpreting the findings of the reviewed studies, potentially introducing bias. This study lacks long-term follow-up to evaluate the sustainability of the therapeutic effects. Therefore, the findings of this study should only be considered preliminary evidence.

Acupuncture therapy has significant promise as an alternate treatment for relieving PD anxiety in patients. However, additional large-sample, high-quality RCTs are needed to confirm its efficacy and determine which types (e.g., electroacupuncture, moxibustion, and auricular acupuncture) and dosages (e.g., frequency and duration of therapy) are most helpful to patients. Additionally, future RCTs should incorporate a follow-up period of at least 3 months to evaluate the sustainability of therapeutic effects. The optimal acupuncture regimen remains to be determined based on current evidence. They highlight directions for future research, such as designing more refined clinical trials to validate the efficacy of different types of acupuncture. Future studies should adopt sham-needle blinding and third-party blinding to minimize measurement bias and implementation bias.

## 5 Conclusion

Preliminary evidence suggests that acupuncture may be effective in alleviating anxiety symptoms in patients with PD. Furthermore, in clinical practice, acupuncture is regarded as a safe technique that may be useful as an alternative or adjunctive treatment. Additional higher quality randomized controlled trials are required to ascertain the safety and effectiveness of acupuncture as a therapy for anxiety in PD patients.

## Data Availability

The original contributions presented in the study are included in the article/Supplementary material, further inquiries can be directed to the corresponding authors.

## References

[ref1] Aarsland D. Batzu L. Halliday G. M. Geurtsen G. J. Ballard C. Ray C. K. . (2021). Parkinson disease-associated cognitive impairment. Nat. Rev. Dis. Primers 7:47. doi: 10.1038/s41572-021-00280-3 34210995

[ref2] Ascherio A. Schwarzschild M. A. (2016). The epidemiology of Parkinson's disease: risk factors and prevention. Lancet Neurol. 15, 1257–1272. doi: 10.1016/S1474-4422(16)30230-7, PMID: 27751556

[ref3] Bai Y. Wang M. (2021). Clinical observation on 56 cases of acupuncture treatment for Parkinson's disease patients with insomnia. Chin. J. Tradit. Med. Sci. Technol. 28, 506–507.

[ref4] Ben-Shlomo Y. Darweesh S. Llibre-Guerra J. Marras C. San L. M. Tanner C. (2024). The epidemiology of Parkinson's disease. Lancet 403, 283–292. doi: 10.1016/S0140-6736(23)01419-8 38245248 PMC11123577

[ref5] Broen M. P. Narayen N. E. Kuijf M. L. Dissanayaka N. N. Leentjens A. F. (2016). Prevalence of anxiety in Parkinson's disease: a systematic review and meta-analysis. Mov. Disord. 31, 1125–1133. doi: 10.1002/mds.26643, PMID: 27125963

[ref6] Carey G. Görmezoğlu M. de Jong J. J. A. Hofman P. A. M. Backes W. H. Dujardin K. . (2021). Neuroimaging of anxiety in Parkinson's disease: a systematic review. Mov. Disord. 36, 327–339. doi: 10.1002/mds.28404, PMID: 33289195 PMC7984351

[ref7] Chae Y. Yeom M. Han J. Park H. Hahm D. Shim I. . (2008). Effect of acupuncture on anxiety-like behavior during nicotine withdrawal and relevant mechanisms. Neurosci. Lett. 430, 98–102. doi: 10.1016/j.neulet.2007.10.026, PMID: 18060697

[ref8] Chaudhuri K. R. Healy D. G. Schapira A. H. (2006). Non-motor symptoms of Parkinson's disease: diagnosis and management. Lancet Neurol. 5, 235–245. doi: 10.1016/S1474-4422(06)70373-8, PMID: 16488379

[ref9] Chen T. Zhang W. W. Chu Y. X. Wang Y. Q. (2020). Acupuncture for pain management: molecular mechanisms of action. Am. J. Chin. Med. 48, 793–811. doi: 10.1142/S0192415X20500408, PMID: 32420752

[ref10] Cho K. Kim T. Kwon S. Jung W. Moon S. Ko C. . (2018). Complementary and alternative medicine for idiopathic Parkinson’s disease: an evidence-based clinical practice guideline. Front. Aging Neurosci. 10:323. doi: 10.3389/fnagi.2018.00323, PMID: 30374299 PMC6196228

[ref11] Cook S. C. Schwartz A. C. Kaslow N. J. (2017). Evidence-based psychotherapy: advantages and challenges. Neurotherapeutics 14, 537–545. doi: 10.1007/s13311-017-0549-4, PMID: 28653278 PMC5509639

[ref12] Deuel L. M. Seeberger L. C. (2020). Complementary therapies in Parkinson disease: a review of acupuncture, tai chi, qi gong, yoga, and Cannabis. Neurotherapeutics 17, 1434–1455. doi: 10.1007/s13311-020-00900-y, PMID: 32785848 PMC7851283

[ref13] Dissanayaka N. N. Sellbach A. Matheson S. O'Sullivan J. D. Silburn P. A. Byrne G. J. . (2010). Anxiety disorders in Parkinson's disease: prevalence and risk factors. Mov. Disord. 25, 838–845. doi: 10.1002/mds.22833, PMID: 20461800

[ref14] Ehgoetz Martens K. A. Szeto J. Y. Muller A. J. Hall J. M. Gilat M. Walton C. C. . (2016). Cognitive function in Parkinson's disease patients with and without anxiety. Neurol. Res. Int. 2016:6254092. doi: 10.1155/2016/6254092, PMID: 27800180 PMC5075302

[ref15] Fan J. Q. Lu W. J. Tan W. Q. Liu X. Wang Y. T. Wang N. B. . (2022). Effectiveness of acupuncture for anxiety among patients with Parkinson disease: a randomized clinical trial. JAMA Netw. Open 5:e2232133. doi: 10.1001/jamanetworkopen.2022.32133, PMID: 36129711 PMC9494193

[ref16] GBD 2016 Parkinson's Disease Collaborators (2018). Global, regional, and national burden of Parkinson's disease, 1990-2016: a systematic analysis for the global burden of disease study 2016. Lancet Neurol. 17, 939–953. doi: 10.1016/S1474-4422(18)30295-3, PMID: 30287051 PMC6191528

[ref17] Gros P. Videnovic A. (2020). Overview of sleep and circadian rhythm disorders in Parkinson disease. Clin. Geriatr. Med. 36, 119–130. doi: 10.1016/j.cger.2019.09.005, PMID: 31733692 PMC6921931

[ref18] Guyatt G. H. Oxman A. D. Vist G. E. Kunz R. Falck-Ytter Y. Alonso-Coello P. . (2008). GRADE: an emerging consensus on rating quality of evidence and strength of recommendations. BMJ 336, 924–926. doi: 10.1136/bmj.39489.470347 18436948 PMC2335261

[ref19] Heneghan C. Mahtani K. R. Goldacre B. Godlee F. Macdonald H. Jarvies D. (2017). Evidence based medicine manifesto for better healthcare. BMJ 357:j2973. doi: 10.1136/bmj.j2973, PMID: 28634227

[ref20] Higgins J. P. Altman D. G. Gøtzsche P. C. Jüni P. Moher D. Oxman A. D. . (2011). The cochrane collaboration's tool for assessing risk of bias in randomised trials. BMJ 343:d5928. doi: 10.1136/bmj.d5928 22008217 PMC3196245

[ref21] Higgins J. P. Thompson S. G. Deeks J. J. Altman D. G. (2003). Measuring inconsistency in meta-analyses. BMJ 327, 557–560. doi: 10.1136/bmj.327.7414.557, PMID: 12958120 PMC192859

[ref22] Höglinger G. U. Adler C. H. Berg D. Klein C. Outeiro T. F. Poewe W. . (2024). A biological classification of Parkinson's disease: the SynNeurGe research diagnostic criteria. Lancet Neurol. 23, 191–204. doi: 10.1016/S1474-4422(23)00404-0, PMID: 38267191

[ref23] Iranzo A. Cochen D. C. V. Fantini M. L. Pérez-Carbonell L. Trotti L. M. (2024). Sleep and sleep disorders in people with Parkinson's disease. Lancet Neurol. 23, 925–937. doi: 10.1016/S1474-4422(24)00170-4 38942041

[ref24] Jellinger K. A. (2022). Morphological basis of Parkinson disease-associated cognitive impairment: an update. J. Neural Transm. 129, 977–999. doi: 10.1007/s00702-022-02522-4, PMID: 35726096

[ref25] Kwok J. Kwan J. Auyeung M. Mok V. Lau C. Choi K. C. . (2019). Effects of mindfulness yoga vs stretching and resistance training exercises on anxiety and depression for people with Parkinson disease: a randomized clinical trial. JAMA Neurol. 76, 755–763. doi: 10.1001/jamaneurol.2019.0534, PMID: 30958514 PMC6583059

[ref26] Li J. L. Shi J. (2025). Efficacy study on integrated Chinese-western therapy in treatment of Parkinson’s disease with liver and kidney deficiency syndrome. J. Hubei Univ. Chin. Med. 27, 61–63.

[ref27] Li L. Tian Z. W. Zhang X. (2021). Clinical study on Electroacupuncture combined with Madopar for sleep disorders in Parkinson disease. New Chin. Med. 53, 113–116. doi: 10.13457/j.cnki.jncm.2021.01.030

[ref28] Li K. Xu S. Wang R. Zou X. Liu H. Fan C. . (2023). Electroacupuncture for motor dysfunction and constipation in patients with Parkinson's disease: a randomised controlled multi-Centre trial. EClinicalMedicine 56:101814. doi: 10.1016/j.eclinm.2022.101814, PMID: 36691434 PMC9860357

[ref29] Lin S. S. Zhou B. Chen B. J. Jiang R. T. Li B. Illes P. . (2023). Electroacupuncture prevents astrocyte atrophy to alleviate depression. Cell Death Dis. 14:343. doi: 10.1038/s41419-023-05839-4, PMID: 37248211 PMC10227075

[ref30] Liu L. (2020). Clinical efficacy of duloxetine combined with acupuncture in the treatment of parkinsonian anxiety. Inner Mongolia Tradit. Chin. Med. 39, 137–138. doi: 10.16040/j.cnki.cn15-1101.2020.08.086

[ref31] Lu W. (2022). Clinical observation of regulating Spiriy acupuncture technique in treatment of Parkinson disease anxiety. Guangzhou Univ. Chin. Med. 2022, 1–6. doi: 10.27044/d.cnki.ggzzu.2022.000503

[ref32] Lu P. Fang M. Yao L. Zhang N. Xu K. He P. (2022). Massage of bladder Meridian relieved anxiety induced by chronic stress in rats. Biomed. Res. Int. 2022:5639716. doi: 10.1155/2022/5639716, PMID: 36531656 PMC9754834

[ref33] Ma J. Li L. Teng Y. (2024). Effect of empathy nursing combined with acupoint application on anxiety symptoms in patients with Parkinson’s disease accompanied by anxiety. J. Clin. Nurs. Pract. 10, 45–48. doi: 10.11997/nitcwm.202405012

[ref34] Mak M. K. Wong-Yu I. S. Shen X. Chung C. L. (2017). Long-term effects of exercise and physical therapy in people with Parkinson disease. Nat. Rev. Neurol. 13, 689–703. doi: 10.1038/nrneurol.2017.128, PMID: 29027544

[ref35] Martens K. Hall J. M. Gilat M. Georgiades M. J. Walton C. C. Lewis S. (2016). Anxiety is associated with freezing of gait and attentional set-shifting in Parkinson's disease: a new perspective for early intervention. Gait Posture 49, 431–436. doi: 10.1016/j.gaitpost.2016.07.182, PMID: 27513741

[ref36] Mirelman A. Bonato P. Camicioli R. Ellis T. D. Giladi N. Hamilton J. L. . (2019). Gait impairments in Parkinson's disease. Lancet Neurol. 18, 697–708. doi: 10.1016/S1474-4422(19)30044-4, PMID: 30975519

[ref37] Noh H. Kwon S. Cho S. Jung W. Moon S. Park J. . (2017). Effectiveness and safety of acupuncture in the treatment of Parkinson’s disease: a systematic review and meta-analysis of randomized controlled trials. Complement. Ther. Med. 34, 86–103. doi: 10.1016/j.ctim.2017.08.005, PMID: 28917379

[ref38] Opara J. A. Brola W. Leonardi M. Błaszczyk B. (2012). Quality of life in Parkinson's disease. J. Med. Life 5, 375–381. 23346238 PMC3539848

[ref39] Pachana N. A. Egan S. J. Laidlaw K. Dissanayaka N. Byrne G. J. Brockman S. . (2013). Clinical issues in the treatment of anxiety and depression in older adults with Parkinson's disease. Mov. Disord. 28, 1930–1934. doi: 10.1002/mds.25689, PMID: 24123116

[ref40] Page M. J. McKenzie J. E. Bossuyt P. M. Boutron I. Hoffmann T. C. Mulrow C. D. . (2021). The PRISMA 2020 statement: an updated guideline for reporting systematic reviews. BMJ 372:n71. doi: 10.1136/bmj.n71, PMID: 33782057 PMC8005924

[ref41] Reijnders J. S. Ehrt U. Weber W. E. Aarsland D. Leentjens A. F. (2008). A systematic review of prevalence studies of depression in Parkinson's disease. Mov. Disord. 23:quiz 313, 183–189. doi: 10.1002/mds.21803 17987654

[ref42] Salanti G. Ades A. E. Ioannidis J. P. A. (2011). Graphical methods and numerical summaries for presenting results from multiple-treatment meta-analysis: an overview and tutorial. J. Clin. Epidemiol. 64, 163–171. doi: 10.1016/j.jclinepi.2010.03.016, PMID: 20688472

[ref43] Schapira A. Chaudhuri K. R. Jenner P. (2017). Non-motor features of Parkinson disease. Nat. Rev. Neurosci. 18, 435–450. doi: 10.1038/nrn.2017.62, PMID: 28592904

[ref44] Song L. J. (2021). Effects of acupuncture and moxibustion combined with hyperbaric oxygen and rehabilitation exercise on non-motor symptoms and mental state of patients with Parkinson's disease. Reflexol. Rehabil. Med. 2:3.

[ref45] Sterne J. Savović J. Page M. J. Elbers R. G. Blencowe N. S. Boutron I. . (2019). RoB 2: a revised tool for assessing risk of bias in randomised trials. BMJ 366:l4898. doi: 10.1136/bmj.l4898, PMID: 31462531

[ref46] Tan E. K. Chao Y. X. West A. Chan L. L. Poewe W. Jankovic J. (2020). Parkinson disease and the immune system - associations, mechanisms and therapeutics. Nat. Rev. Neurol. 16, 303–318. doi: 10.1038/s41582-020-0344-4, PMID: 32332985

[ref47] Tansey M. G. Wallings R. L. Houser M. C. Herrick M. K. Keating C. E. Joers V. (2022). Inflammation and immune dysfunction in Parkinson disease. Nat. Rev. Immunol. 22, 657–673. doi: 10.1038/s41577-022-00684-6, PMID: 35246670 PMC8895080

[ref48] Tolosa E. Garrido A. Scholz S. W. Poewe W. (2021). Challenges in the diagnosis of Parkinson's disease. Lancet Neurol. 20, 385–397. doi: 10.1016/S1474-4422(21)00030-2, PMID: 33894193 PMC8185633

[ref49] Tolosa E. Wenning G. Poewe W. (2006). The diagnosis of Parkinson's disease. Lancet Neurol. 5, 75–86. doi: 10.1016/S1474-4422(05)70285-4, PMID: 16361025

[ref50] Tysnes O. B. Storstein A. (2017). Epidemiology of Parkinson's disease. J. Neural Transm. 124, 901–905. doi: 10.1007/s00702-017-1686-y, PMID: 28150045

[ref51] Vijiaratnam N. Simuni T. Bandmann O. Morris H. R. Foltynie T. (2021). Progress towards therapies for disease modification in Parkinson's disease. Lancet Neurol. 20, 559–572. doi: 10.1016/S1474-4422(21)00061-2, PMID: 34146514

[ref52] Weintraub D. Aarsland D. Chaudhuri K. R. Dobkin R. D. Leentjens A. F. Rodriguez-Violante M. . (2022). The neuropsychiatry of Parkinson's disease: advances and challenges. Lancet Neurol. 21, 89–102. doi: 10.1016/S1474-4422(21)00330-6, PMID: 34942142 PMC8800169

[ref53] White A. Cummings M. Barlas P. Cardini F. Filshie J. Foster N. E. . (2008). Defining an adequate dose of acupuncture using a neurophysiological approach--a narrative review of the literature. Acupunct. Med. 26, 111–120. doi: 10.1136/aim.26.2.111, PMID: 18591910

[ref54] Xu Z. Xia J. (2017). The effect of auricular acupuncture point burying beans combined with psychological care on anxiety and depression in patients with Parkinson's disease. Massage Rehabil. Med. 8, 73–74. doi: 10.19787/j.issn.1008-1879.2017.01.037

[ref55] Xu Y. Zhu X. Chen Y. Chen Y. Zhu Y. Xiao S. . (2023). Electroacupuncture alleviates mechanical allodynia and anxiety-like behaviors induced by chronic neuropathic pain via regulating rostral anterior cingulate cortex-dorsal raphe nucleus neural circuit. CNS Neurosci. Ther. 29, 4043–4058. doi: 10.1111/cns.14328, PMID: 37401033 PMC10651964

[ref56] Zheng J. Y. Zhu J. Wang Y. Tian Z. Z. (2024). Effects of acupuncture on hypothalamic-pituitary-adrenal axis: current status and future perspectives. J. Integr. Med. 22, 445–458. doi: 10.1016/j.joim.2024.06.004, PMID: 38955651

[ref57] Zhu J. Y. Gao X. Y. Wang L. L. Ren Z. X. Wang G. L. Guo J. . (2025). A randomized controlled trial on the clinical efficacy and safety of acupuncture combined with levodopa in the treatment of Parkinson's disease. J. Tradit. Chin. Med. 66, 1456–1462. doi: 10.13288/j.11-2166/r.2025.14.009

