# Supplementary material for: Effectiveness and safety of acupuncture for Parkinson’s disease anxiety: a systematic review and meta-analysis
Source: Front Aging Neurosci. 2025 Oct 15;17:1663059. doi: 10.3389/fnagi.2025.1663059 (PMC12568604; doi:10.3389/fnagi.2025.1663059)
Supplement: Supplementary file 1 [file Data_Sheet_1.docx]

| Database |  |
| --- | --- |
| CNKI | SU%=(‘帕金森’ + ‘帕金森病’ + ‘帕金森综合征’）*（‘非运动症状' + ‘焦虑')*(‘针灸' + ‘电针' + ‘手工针灸' + ‘艾灸' + ‘穴位埋线' + ‘经皮电刺激' + ‘穴位敷贴' + ‘穴位按摩' + ‘耳穴压豆' + ‘耳穴') |
| Wanfang | 主题:(((“帕金森” or “帕金森病” or “帕金森综合征”) and (“非运动症状” or “焦虑”)) and (“针灸” or “电针” or “手工针灸” or “艾灸” or “穴位埋线” or “经皮电刺激” or “穴位敷贴” or “穴位按摩” or “耳穴压豆” or “耳穴”) |
| VIP | M=(((“帕金森” or “帕金森病” or “帕金森综合征”) and (“非运动症状” or “焦虑”)) and (“针灸” or “电针” or “手工针灸” or “艾灸” or “穴位埋线” or “经皮电刺激” or “穴位敷贴” or “穴位按摩” or “耳穴压豆” or “耳穴”)) |
| CBM | (("帕金森"[常用字段:智能] AND ("焦虑"[常用字段:智能] OR "非运动症状"[常用字段:智能])) AND （"针灸"[常用字段:智能] OR "电针"[常用字段:智能] OR "针灸"[常用字段:智能] OR "艾灸"[常用字段:智能] OR "穴位敷贴"[常用字段:智能] OR "耳穴压豆"[常用字段:智能])) |
| PubMed | ((“acupuncture” OR “electroacupuncture” OR “manual acupuncture” OR “moxibustion” OR “acupoint catgut embedding” OR “transcutaneous electrical acupoint stimulation” OR “acupressure” [Mesh]) AND ("Parkinson Disease" [Mesh]) AND (“anxiety OR “non-motor symptoms” [Mesh])) |
| Embase | ('acupuncture':ab,ti OR 'electroacupuncture':ab,ti OR 'moxibustion':ab,ti OR 'manual acupuncture':ab,ti OR 'catgut embedding':ab,ti OR 'transcutaneous electrical acupoint stimulation':ab,ti OR 'acupressure':ab,ti) AND ('non-motor symptoms':ab,ti OR 'anxiety':ab,ti) AND ('Parkinson Disease':ab,ti) |
| Cochrane Library | #1 (“acupuncture” OR “electroacupuncture” OR “manual acupuncture” OR “moxibustion” OR “acupoint catgut embedding” OR “transcutaneous electrical acupoint stimulation” OR “acupressure”):ti,ab,kw  #2 (“Parkinson Disease”):ti,ab,kw  #3("anxiety" OR "non-motor symptoms"):ti,ab,kw  #1 and #2 and #3 |
| Web of Science | TS= ((‘acupuncture’ OR ‘electroacupuncture’ OR ‘manual acupuncture’ OR ‘moxibustion’ OR ‘acupoint catgut embedding’ OR ‘transcutaneous electrical acupoint stimulation’ OR ‘acupressure’) AND (‘Parkinson Disease’) AND ('anxiety' OR ‘non-motor symptoms’) ) |
| Scopus | TITLE-ABS-KEY ( "acupuncture" OR "electroacupuncture" OR "manual acupuncture" OR "moxibustion" OR "acupoint catgut embedding" OR "transcutaneous electrical acupoint stimulation" OR "acupressure") AND TITLE-ABS-KEY ( "Parkinson Disease" OR "PD") AND TITLE-ABS ( "anxiety" OR "non-motor symptoms") |

Table S1. Search Strategies for Each Database

CBM: China Biology Medicine; CNKI: China National Knowledge Infrastructure; Embase: Excerpta Medica Database; VIP: VIP Database for Chinese Technical Periodicals.
